# AB209630, a long non-coding RNA decreased expression in hypopharyngeal squamous cell carcinoma, influences proliferation, invasion, metastasis, and survival

**DOI:** 10.18632/oncotarget.7403

**Published:** 2016-02-15

**Authors:** Jieyu Zhou, Maocai Li, Wenbin Yu, Wenming Li, Juan Wang, Xuan Xiang, Guojun Li, Xinliang Pan, Dapeng Lei

**Affiliations:** ^1^ Department of Otorhinolaryngology, Qilu Hospital, Shandong University, Key Laboratory of Otolaryngology, Ministry of Health, Shandong, P. R. China; ^2^ Key Laboratory of Carcinogenesis and Translational Research (Ministry of Education/Beijing), Department of Head and Neck surgery, Peking University Cancer Hospital & Institute, Beijing, China; ^3^ Department of Head and Neck Surgery, The University of Texas MD Anderson Cancer Center, Houston, TX, USA

**Keywords:** lncRNA, AB209630, hypopharyngeal cancer, survival, invasion

## Abstract

Long noncoding RNAs (lncRNAs) are associated with the development, progression, and prognosis of human cancers. However, the clinical significance and biological function of lncRNAs in hypopharyngeal squamous cell carcinoma (HSCC) remain largely unknown. We characterized the novel lncRNA AB209630 *in vivo* and *in vitro*. First, using qRT-PCR, we evaluated whether AB209630 levels differ between HSCC tissues/cell lines and adjacent normal tissues/cell lines. We then assessed whether AB209630 expression levels stimulate or inhibit proliferation, invasion, apoptosis, and metastasis *in vitro*. Finally, we investigated whether AB209630 levels in tumor tissues were associated with survival outcomes. Our results demonstrated that AB209630 levels were markedly lower in HSCC tissues and cells than in normal tissues and cells, and increased expression of AB209630 level significantly inhibited growth, metastasis, and invasion and stimulated apoptosis *in vitro*. In addition, patients with decreased expression of AB209630 had a significantly poorer prognosis than those with high AB209630 expression. These data suggest that increased expression of AB209630 might either stimulate or inhibit biological activities involved in HSCC development, indicating a potential application of AB209630 in future treatment for this disease. This study suggest that AB209630 functions as a tumor suppressor in HSCC, and its decreased expression may help predict a poor prognostic outcome of HSCC. Our future work will focus on the mechanisms of whether and how AB209630 as a tumor suppressor gene is involved in HSCC development.

## INTRODUCTION

Hypopharyngeal squamous cell carcinomas (HSCCs) account for approximately 5-15% [1] of all head and neck cancers and have the worst prognosis among squamous cell carcinomas of the head and neck [2]. Currently, the main treatment strategy for HSCC is still surgery followed by radiotherapy. Although the majority of patients with early-stage HSCC can be cured by surgery, more than half of those with advanced-stage disease die of carcinoma recurrence or the occurrence of a second primary malignancy. The 5-year overall survival rate is estimated to be at 25% to 40% [3].

A better understanding of the pathogenesis and molecular alterations of HSCC is essential for development and novel effective therapies of HSCC. Many molecules, such as SIRT1, DBC1 [4], Beclin-1, LC3, [5] and Caveolin-1 [6], have been evaluated as candidate biomarkers for HSCC, but none have been widely used in practice. Because understanding of the molecular mechanisms involved in HSCC development, progression, and treatment response is still lacking; therefore, the identification of novel and more effective molecular biomarkers for HSCC prognosis and progression is necessary.

Long non-coding RNAs (lncRNAs; > 200 nucleotides) are defined as non-protein-coding RNAs distinct from housekeeping RNAs such as tRNAs, rRNAs, and snRNAs [7,8]. LncRNAs is involved in in almost every aspect of cell biology, including chromosome remodeling, transcription, and post-transcriptional processing [9–12]. Therefore, aberrant lncRNA expression can cause various human diseases, including cancers [12,13]. Aberrant expression of lncRNAs has been demonstrated in various human solid tumors, such as prostate cancer, non-small cell lung cancer, liver cancer, breast cancer, colorectal cancer, laryngeal cancer, and testicular cancer [14–20]. Several lncRNAs have recently been discovered in cancers [21], but the correlations between aberrant expression of certain lncRNAs and biological function and outcomes of HSCC have not been characterized.

In the present study, we aimed to identify new lncRNAs involved in HSCC and test whether the expression of those lncRNAs could be a potential biomarker for HSCC prognosis. Specifically, we first identified all aberrantly expressed lncRNAs in HSCC using microarray assays. Second, we investigated the expression patterns and genomic locations of the aberrantly expressed lncRNAs. Among them, two lncRNAs (down-expressed AB209630 and up-expressed AB019562) were selected and their expression patterns were confirmed by quantitative real-time polymerase chain reaction (qRT-PCR). Finally, we explored correlations of expression changes of these lncRNAs with biological functions and survival of HSCC patients. In this study we firstly selected AB209630 for study since AB209630 ranked one of top 3 miRNAs with most significant changes in expression in HSCC and its function remained unknown.

## RESULTS

### LncRNA expression in three paired human samples

For the global profiling of human lncRNA expression, we analyzed three paired primary cancerous and adjacent noncancerous tissue samples using the Arraystar Human LncRNA Microarray and detected 33,045 lncRNAs. By comparing the transcriptome sequences of the cancer tissues with those of their matched normal tissues, we identified significant differential expression of two lncRNAs (AB209630 and AB019562). AB209630 had decreased expression, whereas AB019562 was highly expressed in HSCC.

### Confirmation of differential expression of AB209630 and AB019562 by qRT-PCR

**Figure 1:**
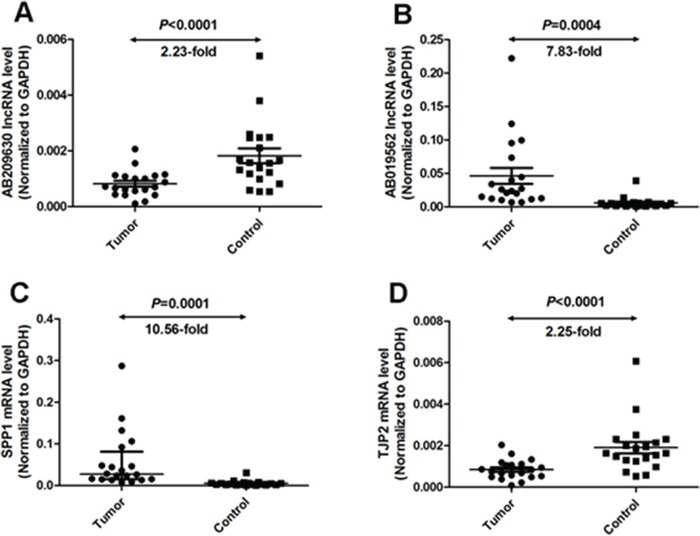
Quantitative determination of lncRNA/mRNA by means of qRT-PCR for AB209630 **(A)**, AB019562 **(B)**, SPP1 **(C)**, and TJP2 **(D)** in tissues from hypopharyngeal carcinomas and adjacent noncancerous mucosae RNA relative expression levels were given as ratios of GAPDH transcript levels in the same RNA sample. Scatter plots are shown with medians with interquartile range. Statistical analyses were performed with the Wilcoxon matched paired test (2-tailed).

To confirm the reliability and the validity of the microarray hybridization procedure, the expression levels of the two identified lncRNAs (AB209630 and AB019562) were analyzed by qRT-PCR in 20 HSCC tumors and matched non-cancerous mucosal epithelial tissue samples. The relative expression levels of study RNA were given as ratios of GAPDH transcript levels in the same RNA sample. Our qRT-PCR assays showed that AB209630 expression decreased significantly in carcinomas compared with adjacent noncancerous mucosae (*P*<0.0001, 2.23-fold, Figure 1A), while AB019562 expression was significantly higher in carcinomas than in adjacent noncancerous mucosae (*P*=0.0004, 7.83-fold, Figure 1B). These microarray results were further confirmed using 2 mRNAs. SPP1 (Figure 1C) and TJP2 (Figure 1D).

All of the 20 patients these samples were obtained from were male, with a median age of 58.5 years (range, 39–73). Almost half had advanced T-stage disease (T3–T4 in 45.0%), and most had lymph node metastasis (85.0%) and advanced overall stage disease (95.0% stage III/IV) cancers. All subjects were smokers or drinkers. All patients had pathologically confirmed diagnoses and were staged according to the International Union against Cancer (UICC, 2002) TNM classification.

### Increased expression of AB209630 by AB209630-expressing lentiviral vector

**Figure 2:**
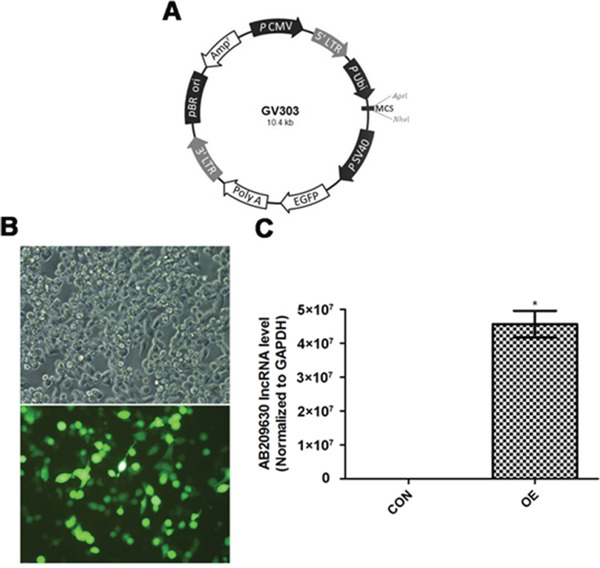
AB209630 was successfully upregulated by AB209630-expressing lentiviral vector **A.** Schematic diagram of plasmid. **B.** The transfected 293T cells were observed under a fluorescence microscope after 48h (×200). **C.** Quantitative real-time PCR was performed to measure the expression of AB209630 in 293T cells (CON) and lentivirus-infected cells (OE). Results are mean ± SD (n=3; *P=0.002).

To investigate the biological significance of AB209630 in FaDu cells, we constructed an AB209630 expression lentiviral vector (Figure 2A), and lentiviruses were produced by transient transfection of HEK293T cells (Figure 2B). We transfected the AB209630 expression lentiviral vector into FaDu cells for overexpression (OE); stably transfected the FaDu cells with the same empty lentiviral vector as a negative control (NC); and used parental FaDu cells as a control (CON). Through RNA expression analysis, we found that AB209630 expression in the OE cells was significantly higher than in either the CON cells (Figure 2C). The successful establishment of an AB209630 overexpression FaDu cell clone provided a useful tool for investigating the function of AB209630 in FaDu cells.

### Inhibition of FaDu cell growth by AB209630 overexpression

**Figure 3:**
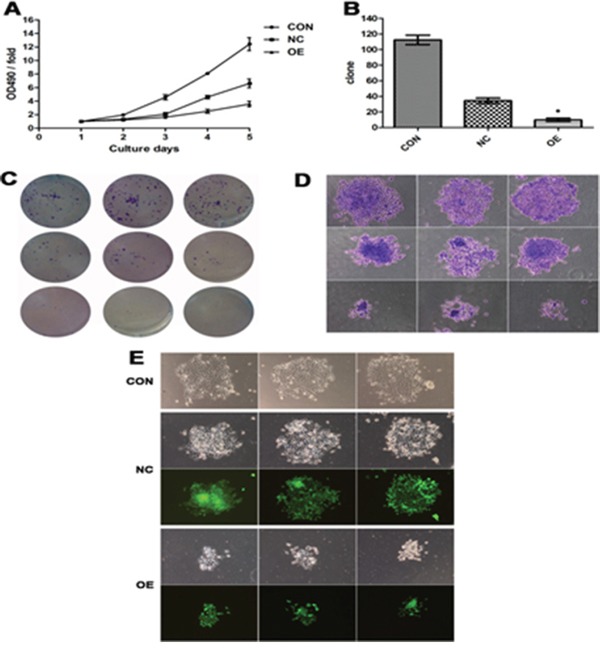
Increased expression of AB209630 suppressed the proliferation of FaDu cells **A.** The proliferation assay was performed on parental (CON), lentiviral vector control (NC), and overexpression lncRNA-AB209630 (OE) FaDU cells. The absorbance was measured on days 1, 2, 3, 4, and 5 according to the MTT method. **B.** The Gimsa-stained colonies were observed and measured under a microscope (×200). A bar graph shows the differences in colony formation among the three groups. The data are presented as the mean ± SD for three independent experiments (*P<0.01, compared with NC). **C.** Colonies were photographed. **D.** The diameter of OE sarcospheres was much smaller than that of CON and NC sarcospheres. **E.** Sarcospheres were photographed under a fluorescent microscope (×200).

As shown in Figure 3A, the OE FaDu cells had significantly slower growth than the two control cell lines (*P*<0.05), which suggests that AB209630 inhibits the growth of FaDu cells. Increased expression of AB209630 in FaDu cells also inhibited proliferation, as shown in the colony formation assay. After 2 weeks, the NC cell colony grew by 34 ± 4, and the OE cell colony grew by only 10 ± 2 (*P* < 0.01) (Figure 3B, 3C). Moreover, OE cells had smaller sarcospheres compared with CON and NC cells (Figure 3D, 3E). Therefore, the low cell viability detected by MTT assay and the low number of cell colonies from OE cells demonstrated that increased expression of AB209630 inhibits the growth of FaDu cells.

### Effect of increased expression of AB209630 on FaDu cell death

**Figure 4:**
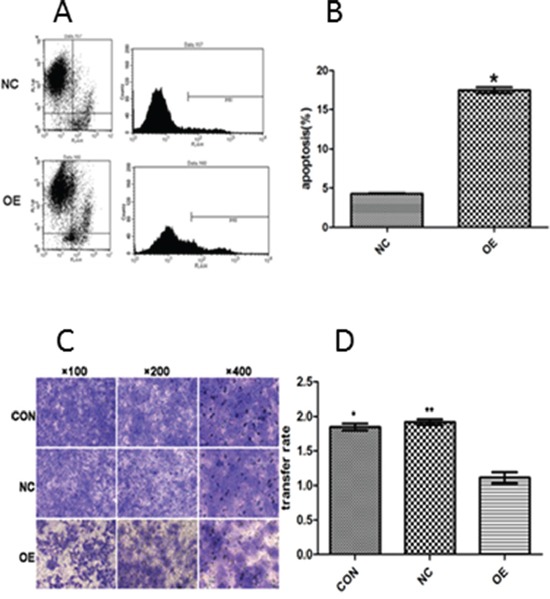
The apoptosis rate was evaluated quantitatively by flow cytometry (A and B) and the suppressive effect of AB209630 on the invasion of FaDu cells (C and D) **A.** The figure shows a representative result; **B.** The comparison of apoptosis rates between NC and OE cells. The data are presented as the mean ± SD for three independent experiments (*P<0.01); **C.** Transwell invasion assay showed that increased AB209630 expression inhibited cell invasion of FaDu cells. The Gimsa-stained photographs were observed and measured under a microscope (×100, ×200, and ×400). Representative of three experiments; and **D.** Graphical representation of the rates of invading FaDu cells in the three groups. Data are shown as mean ± SD from three experiments (*P<0.05, CON versus OE; **P<0.05, NC versus OE).

We then examined the impact of AB209630 overexpression on apoptosis. Measurement of Annexin V-APC by FACS analysis showed that the proportion of the cell population undergoing apoptosis was 4.31±0.06% for NC cells and 17.48±0.39% for OE cells. These results suggest that increased expression of AB209630 significantly increased cell death or apoptosis in FaDu cells but not in NC cells (*P* < 0.001) (Figure 4A, 4B).

### Effect of increased expression of AB209630 on invasion of FaDu cells

We further examined whether increased expression of AB209630 could affect the invasiveness of FaDu cells *in vitro*. The Transwell invasion assay showed that increased expression of AB209630 in FaDu cells resulted in markedly decreased invasive behavior, as indicated by a significant decrease in the average number of OE cells that invaded through the Matrigel compared with the CON or NC cells (*P*<0.05) (Figure 4C, 4D). These results indicated that increased expression of AB209630 may inhibit invasiveness of FaDu cells.

### Association of AB209630 expression with survival of HSCC patients

**Figure 5:**
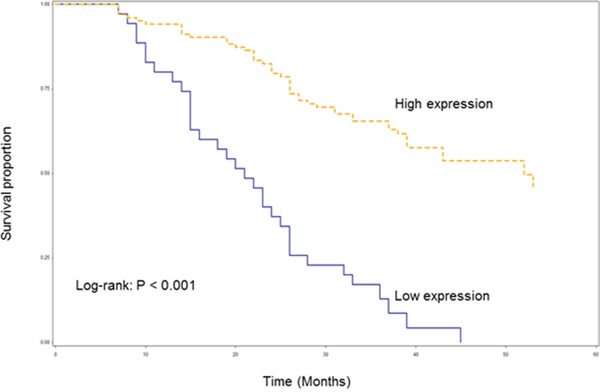
Kaplan-Meier overall survival curve stratified by lncRNA *AB209630* expression

Among 138 patients with HSCC, the median follow-up period for these patients was 33 months (range, 7-53 months). Of these patients, a total of 78 patients died, mainly owing to disease recurrence and second primary malignancy followed in frequency by death from causes unrelated to cancer. As shown in Figure 5, the difference in OS between HSCC patients with high and low expression of AB209630 was statistically significant (log-rank: *P* < 0.001). The HSCC patients with increased expression of AB209630 had significantly better OS than those with decreased expression of AB209630. Multivariable analysis showed that patients with increased expression of AB209630 had significantly reduced risk of death overall compared to those with decreased expression of AB209630 (HR, 0.25; 95% CI, 0.15-0.42) (Table 1).

**Table 1:** Multivariable survival analysis by AB209630 expression in 138 HSCC patients

AB209630 expression	Total (138)	Death (all causes) N (%)	Survival (OS)	
			Crude HR, 95% CI	Adjusted HR*, 95% CI
Low	35	33 (42.3)	1.00	1.00
High	103	45 (57.7)	0.21 (0.13-0.34)	0.25 (0.15-0.42)

* Adjusted for age, sex, smoking, alcohol, overall stage, tumor sites, differentiation, T, N, node metastasis, and treatment in Cox models.

## DISCUSSION

Approximately 20,000 lncRNAs are estimated to be in the human transcriptome [21]. Although some lncRNA transcripts may represent transcriptional noise, lncRNAs have critical and broad regulation in biological activities, including post-transcriptional regulation, post-translational regulation of protein activity, organization of protein complexes, cell–cell signaling, and allosteric regulation of proteins [22–26]. Dysregulation of gene expression is critical for carcinogenesis and metastasis including protein-coding and non-protein-coding genes [27,28]. Recently, the underlying molecular mechanisms of some miRNAs as tumor suppressors or tumor promotors through post-transcriptional regulation of protein expression have been well demonstrated [29, 30]. LncRNAs are now emerging as new targets in the cancer paradigm, have regulatory functions in both oncogenic and tumor-suppressive pathways [31–34], and are involved in development of various human cancers [14, 17-20, 35-40].

Recent advances in genome and transcriptome study have enabled identification of numerous cancer-related lncRNAs [41]. In the present study, we used the Arraystar LncRNA Gene Chip technology to identify two lncRNA transcripts with significant differences in expression: AB209630 and AB019562. To further understand the biological function of AB209630 in HSCC, we performed a series of *in vitro* assays. Using a lentiviral vector system, we achieved overexpression of AB209630 in FaDu cells. The increased expression of AB209630 inhibited the growth of FaDu cells, proliferation, and colony formation. Furthermore, we found that increased expression of AB209630 significantly decreased the invasive ability of FaDu cells compared to the CON or NC cells, while increased expression of AB209630 activated apoptosis. These results indicate that AB209630 may have a tumor suppressor-like function in HSCC. We also found that AB209630 might also serve as a prognostic biomarker for HSCC patients. Based on the data from 138 patients, we evaluated the association between AB209630 expression levels and survival in HSCC. To our knowledge, this is the first report on association of AB209630 expression with survival of HSCC. However, the exact mechanisms through which AB209630 are involved in HSCC need further investigation.

The use of noncoding RNAs in diagnostics has intrinsic advantages over that of protein-coding RNAs since mature lncRNA is the functional end-product [42]. Therefore, measurement of its expression directly represents the levels of the active molecule [42]. Moreover, lncRNAs are typically more cell-type specific than protein-coding genes [43] and may allow estimation of the cellular composition of a tumor by marking a specific cell population [11]. Many lncRNAs are expressed in a tissue- and cancer-type-restricted manner and have already been shown to be useful as prognostic markers, such as HOTAIR in breast tumors and hepatocellular carcinomas [16, 44], MALAT1 in non-small cell lung cancer [38]. In this study we found that AB209630 expression could be predictive for prognosis of HSCC patients, while information on smoking and alcohol use should be included for adjustment for future prognosis analysis. Moreover, we will confirm our results in other HSCC cell lines in our future studies once they become available. More importantly, investigation on the mechanisms underlying how AB209630 functions as a tumor suppressor gene in HSCC is warranted.

## MATERIALS AND METHODS

### Patients and tissue specimens

We retrospectively analyzed tissue samples and patient data from patients who had undergone surgical treatment for primary HSCC at Qilu Hospital of Shandong University, Jinan, China. All patients had a pathological diagnosis of HSCC before surgery. Primary tumor subsite, clinical stage, treatment, and vital status were recorded from the medical records. Patients who had received neoadjuvant chemotherapy or radiation therapy before surgery were excluded from this study. Between November 2012 and April 2013, three paired primary cancerous and adjacent noncancerous tissue samples were used for global profiling of human lncRNA expression using the Arraystar Human lncRNA Microarray (Arraystar, Rockville, MD, USA). Additionally, 20 HSCC specimens and their matched non-cancerous mucosal epithelial tissues were obtained for confirmation of differential lncRNA expression by qRT-PCR. Between 2009 and 2011, tissue samples and medical records for 138 HSCC patients were obtained and then followed for the survival analysis. The study protocol was approved by the institutional review board (IRB) of the Ethics Boards of Qilu Hospital (the permit numbers is 12040), and tissue specimen acquisition was carried out in accordance with institutional guidelines. All subjects signed written informed consent, and this consent procedure was approved by the IRB of the Ethics Boards of Qilu Hospital.

### RNA extraction and microarray hybridization

Fresh specimens had been stored immediately in liquid nitrogen for total RNA extraction. Total RNA was extracted from each sample using TRIzol reagent (Invitrogen, Carlsbad, CA) following the manufacturer's instructions. RNA concentration was quantified by NanoDrop ND-1000 (NanoDrop Technologies/Thermo Scientific, Wilmington, DE), and RNA integrity was assessed by standard denaturing agarose gel electrophoresis. RNA from the three paired specimens was employed for microarray analysis. Sample preparation and microarray hybridization were performed based on the manufacturer's standard protocols, with minor modifications. Briefly, total RNA was purified after removal of rRNA and tRNA (mRNA-ONLY^TM^ Eukaryotic mRNA Isolation Kit, Epicentre, Madison, WI, USA). Then, each sample was amplified and transcribed into fluorescent cRNA along the entire length of the transcript, without 3′ bias, utilizing a random priming method. The labeled cRNAs were hybridized onto the Human lncRNA Array v2.0 (8 × 60K, Arraystar). After the slides were washed, the arrays were scanned by the Agilent G2505C Scanner (Agilent Technologies, Santa Clara, CA, USA).

### Microarray data analysis

Agilent Feature Extraction software (v11.0.1.1) was used to analyze the acquired array images. Quantile normalization and subsequent data processing were performed using the GeneSpring GX v11.5.1 software package (Agilent Technologies). After quantile normalization of the raw data, lncRNAs and mRNAs that were flagged as Present or Marginal (“All Targets Value”) in all six samples were chosen for further data analysis. Differential expression of lncRNAs and mRNAs that was statistically significant between the two groups was identified through Volcano Plot filtering. Pathway analysis and gene ontology (GO) analysis were applied to determine the roles these differentially expressed RNAs in biological pathways and their associated GO terms. Finally, hierarchical clustering was performed to distinguish between the lncRNA and mRNA expression patterns among the samples.

### Quantitative real-time PCR (qRT-PCR)

The qRT-PCR was used to validate the microarray data. Total RNA was reverse-transcribed to cDNA using PrimeScript Reverse Transcriptase (Takara, Dalian, China) following the manufacturer's protocol. The qRT-PCR was performed using the SYBR Green chemistry in the ABI 7900HT Sequence Detection machine (ABI Applied Biosystems, Foster City, CA). The gene-specific primers used were as follows: SPP1, 5′- ACCTGCCAGCAACCGAAGT-3′ (sense), and 5′- GGTGATGTCCTCGTCTGTAGCA-3′ (antisense); TJP2, 5′- GCAGAGCGAACGAAGAGTATG-3′ (sense), and 5′- ATGACGGGATGTTGATGAGG-3′ (antisense); AB019562, 5′- GGATGTCAGGTCTGCGAAACT-3′ (sense), and 5′- GATAGTGTGGTTTATGGACTGAGGT-3′ (antisense); AB209630, 5′- GGGCTATTGTCCCTAAGTTGAT-3′ (sense), and 5′- TGTCTTGTAGAGCATAAGGAAACC-3′ (antisense); GAPDH, 5′- GGGAAACTGTGGCGTGAT-3′ (sense), and 5′- GAGTGGGTGTCGCTGTTGA-3′ (antisense). PCR was performed in a 10-uL reaction volume and consisted of an initial denaturation step at 95°C for 30 sec followed by amplification with 40 cycles at 95°C for 5 sec and 60°C for 30 sec. The threshold cycle (Ct) was defined as the cycle number at which the fluorescence passed a pre-determined threshold. Both target and reference (GAPDH) genes were amplified in separate wells in triplicate. Gene expression was calculated using the comparative threshold cycle (2^−ΔΔCT^) method.

### Recombinant AB209630 lentiviral vector

To generate an AB209630 expression lentiviral vector, we amplified full-length human AB209630 from MCF7 cDNA using PCR. The AB209630 were cloned into the lentiviral expression plasmid (Genechem Bio, Shanghai, China) following the manufacturer's instructions and confirmed by sequencing. Lentiviruses were produced by transient transfection of HEK293T cells (1×10^5^ cells) with pHelper 1.0, pHelper 2.0, and either Ubi-AB209630-SV40-EGFP or Ubi-MCS-SV40-EGFP (empty) plasmid DNA (AgeI and NheI sites) plus Lipofectamine 2000 (Invitrogen) following the manufacturer's protocol. The lentiviral vector was concentrated by low centrifugation at 6000×g for 16 h and resuspended in 1 ml RPMI 1640 medium after culture for 48 h. The titer of the lentivirus vector in filtered supernatants was estimated by measuring the concentration of HIV p24 gag antigen with an ELISA kit (Perkin-Elmer Life Sciences, Orlando, FL, USA).

### Cell culture and generation of stably transfected cells

The FaDu cell line was purchased from the Type Culture Collection of the Chinese Academy of Sciences (Beijing, China). The cells were cultured at 37°C in 5% CO_2_ and RPMI 1640 medium supplemented with 10% fetal bovine serum (FBS), 2 mM glutamine, 100 units/ml penicillin, and 100 μg/ml streptomycin. Cells at 50%-60% confluence were transfected with the AB209630 expression lentiviral construct in six-well culture plates according to the recombinant lentivirus manufacturer's instructions and viewed under a fluorescent microscope (Leica, Germany).

### Cell proliferation assay

3-(4,5-Dimethylthiazol-2-yl)-2,5-diphenyltetrazolium bromide (MTT) assay (Dingguo Bio, Beijing, China) was employed to generate cell growth curves according to the manufacturer's instructions. Briefly, cells at 2×10^3^/well were cultured in 96-well plates in RPMI 1640 with 10% FBS at 37°C in a humidified 5% CO_2_ atmosphere. Each group of cells was plated in six microwells, and the medium was changed every other day. At 1, 2, 3, 4, and 5 days post-transfection, cell proliferation was measured using an ELISA microplate reader (Biotek ElX800, VT, USA).

### Colony formation assay

The effect of AB209630 overexpression on the colony formation of FaDu cells was analyzed by colony formation assay. Briefly, cultured cells were trypsinized and pipetted with RPMI 1640 supplemented with 10% FBS. Cells (400 cells/well) were cultured in triplicate in six-well plates with 10% FBS-supplemented RPMI 1640 at 37°C in humidified 5% CO_2_, and the medium was changed every 3 days. After 2 weeks, cell colonies were washed twice with phosphate-buffered saline and then fixed with 4% paraformaldehyde for 30 min and stained with Gimsa (ECM550; Chemicon, CA, USA) for 20 min. Individual clones with more than 50 cells were counted. Clone-forming efficiency for each type of cell was calculated according to the following formula: clone forming efficiency = number of colonies/number of inoculated cells ×100%.

### Apoptosis assay

Apoptotic cells were identified by using the Annexin V-APC Apoptosis Detection Kit (88-8007-72; eBioscience, San Diego, CA, USA) according to the manufacturer's instructions. Briefly, cells were harvested and resuspended in 100μl staining buffer containing 5μl Annexin V-APC (20μg/ml) and 5μl propidium iodide (PI) (50μg/ml). Cells stained with Annexin V-APC only, cells stained with PI only, and unstained cells were used as controls. Cells were incubated for 15 min at room temperature in the dark. Flow cytometric analysis was performed using a FACSCalibur (Becton–Dickinson, Franklin Lakes, NJ). Each experiment was repeated at least three times to ensure reproducibility. Annexin V-APC-positive, PI-negative cells were identified as early apoptotic cells. Annexin V-APC- and PI-positive cells were identified as late apoptotic cells or necrotic cells.

### Transwell invasion assay

Transwell chambers (3422; Corning, Tewksbury, MA, USA) were used in this assay. Cells growing in the log phase were treated with trypsin and resuspended as a single-cell solution. Then, the cells were counted, and 1×10^5^ cells in 100 μl of serum-free RPMI 1640 medium were placed into the upper Transwell chamber. The Transwell membrane was coated with Matrigel, and 600 μl of RPMI 1640 medium with 30% FBS was added to the lower compartment. The invasion assay was performed at 37°C with 5% CO_2_ in a tissue culture incubator for 72 h. The cells remaining in the upper chamber were removed using a cotton swab, and the cells that invaded into the lower chamber were fixed in methanol and stained with Gimsa. The cells trapped in the membrane pores or attached to the lower membrane surface were photographed using a × 400 microscope objective. For quantification purposes, 10% acetic acid was added to each well to dissolve Gimsa. The stain intensities were measured as absorbance under 570 nm. Cells (5×10^3^/well) from each group were plated in 96-well plates. MTT assay under 490 nm was employed to measure the stain intensities. Invasion ability for individual type of cells was calculated according to the following formula: transfer rate = Gimsa OD 570/MTT OD 490. Statistical results were obtained from three independent experiments.

### Follow-up of patients

In this study, the primary endpoint for survival analysis was overall death. HSCC patients were typically followed and monitored throughout their treatment and post-treatment courses with regularly scheduled clinical and radiographic examinations. Medical record review for the follow-up statuses of all patients was performed under the direct supervision of the head and neck surgeon. Overall survival (OS) was defined as the time from initial diagnosis until death from any cause or last follow-up. Participants who were alive at the end of the study period or lost to follow-up were censored.

### Statistical analysis

SPSS 18.0 (SPSS Inc., Chicago, IL, USA) and GraphPad Prism 5.0 (GraphPad Software Inc., San Diego, CA, USA) statistical software were employed for the analysis. Data are presented as mean ± SD and were analyzed using Student's t-test. The Wilcoxon matched pairs test was used to compare the RNA expression levels in tumor vs. non-tumor tissues. Survival rates were calculated using the Kaplan–Meier method, and the differences between the survival curves were examined using the log-rank test. Univariate Cox proportional hazards regressions were applied to estimate the individual hazard ratio (HR) for overall survival and adjusted for age, sex, smoking status, alcohol use, overall disease stage, tumor sites, differentiation, TNM classification, and treatment. Patients who had drunk at least one alcoholic beverage/per day for at least 1 year during their lifetime were defined as ever drinkers and those who never had such a pattern of drinking were defined as never drinkers. Patients who had smoked at least 100 cigarettes in their lifetime were defined as ever smokers; otherwise, they were considered never smokers. The significant variables in the univariate analyses (*P*<0.05) were put into the multivariate analysis. The HR with 95% confidence interval was measured to estimate the hazard risk of individual factors. In all analyses, a two-sided *P* value <0.05 was considered statistically significant.

## Abbreviations

HSCC: hypopharyngeal squamous cell carcinomas
lncRNA: long non-coding RNA
qRT-PCR: quantitative real-time polymerase chain reaction
OE: over-expression
NC: negative control
CON: control.
